# Thriving in a salty future: morpho-anatomical, physiological and molecular adaptations to salt stress in alfalfa (*Medicago sativa* L.) and other crops

**DOI:** 10.1093/aob/mcae152

**Published:** 2024-08-31

**Authors:** Xu Liu, J Theo M Elzenga, Jan Henk Venema, Kira J Tiedge

**Affiliations:** Groningen Institute for Evolutionary Life Sciences, University of Groningen, Groningen, The Netherlands; Groningen Institute for Evolutionary Life Sciences, University of Groningen, Groningen, The Netherlands; Groningen Institute for Evolutionary Life Sciences, University of Groningen, Groningen, The Netherlands; Groningen Institute for Evolutionary Life Sciences, University of Groningen, Groningen, The Netherlands

**Keywords:** Alfalfa (*Medicago sativa*), alfalfa–rhizobia symbiosis, crop optimization, root anatomy, salt stress, specialized metabolism

## Abstract

**Background:**

With soil salinity levels rising at an alarming rate, accelerated by climate change and human interventions, there is a growing need for crop varieties that can grow on saline soils. Alfalfa (*Medicago sativa*) is a cool-season perennial leguminous crop, commonly grown as forage, biofuel feedstock and soil conditioner. It demonstrates significant potential for agricultural circularity and sustainability, for example by fixing nitrogen, sequestering carbon and improving soil structures. Although alfalfa is traditionally regarded as a moderately salt-tolerant species, modern alfalfa varieties display specific salt-tolerance mechanisms, which could be used to pave its role as a leading crop able to grow on saline soils.

**Scope:**

Alfalfa’s salt tolerance underlies a large variety of cascading biochemical and physiological mechanisms. These are partly enabled by its complex genome structure and out-crossing nature, but which entail impediments for molecular and genetic studies. This review first summarizes the general effects of salinity on plants and the broad-ranging mechanisms for dealing with salt-induced osmotic stress, ion toxicity and secondary stress. Second, we address the defensive and adaptive strategies that have been described for alfalfa, such as the plasticity of alfalfa’s root system, hormonal crosstalk for maintaining ion homeostasis, spatiotemporal specialized metabolite profiles and the protection of alfalfa–rhizobia associations. Finally, bottlenecks for research of the physiological and molecular salt-stress responses as well as biotechnology-driven improvements of salt tolerance are identified and discussed.

**Conclusion:**

Understanding morpho-anatomical, physiological and molecular responses to salinity is essential for the improvement of alfalfa and other crops in saline land reclamation. This review identifies potential breeding targets for enhancing the stability of alfalfa performance and general crop robustness for rising salt levels as well as to promote alfalfa applications in saline land management.

## INTRODUCTION

Soil salinization has become not only an intractable environmental problem but also one of the main concerns for global socioeconomic development. Over 1 billion hectares of land spreading over more than 100 countries are already salt-affected (Parihar *et al.*, 2015; Shahid *et al.*, 2018; Ivushkin *et al.*, 2019), with the impacted area further expanding due to climate change, rising sea levels and human practices (Smethurst *et al.*, 2008; FAO and ITPS, 2015). Each year, 0.3–1.5 million hectares of arable land have to be taken out of production, and for another 20–46 million hectares the production potential is decreasing due to salinity (FAO and ITPS, 2015). Soil salinization limits crop productivity via its negative impact on soil quality traits such as fertility, biodiversity and structure. To avoid the soil-degrading effect of salinization it is important to know more about its causes, mapping techniques, prevention, mitigation and restoration measures (Stavi *et al.*, 2021). Moreover, salinized soils (generally defined as soils with an electrical conductivity of the saturated paste extract ECe ≥ 4 dS m^−1^) lead to inhibition of seed germination as well as reduction of plant growth, yield and quality, and even plant death (Munns and Tester, 2008; Koevoets *et al.*, 2016), therefore necessitating the development of cultivation strategies and new varieties to stabilize agricultural productivity under saline conditions.

Alfalfa (*Medicago sativa*), also called lucerne and known as the ‘queen of forages’, is widely cultivated in the world, especially in the USA, Argentina and China, with an approximate global production area of 30 million hectares (Latif *et al.*, 2023). Its popularity is mainly due to its high biomass productivity, favourable palatability and digestibility, large protein, vitamin and mineral contents, and its substantial economic value (Shi *et al.*, 2017). It has been developed as a dual-purpose biofuel plant by using the stem as feedstock for biofuel production and the leaves as a protein source for feed and food (Hojilla-Evangelista *et al.*, 2017; Shi *et al.*, 2017). Since alfalfa belongs to the legume family Fabaceae, whose members are able to form symbioses with nitrogen-fixing bacteria, it is also being used as green manure and cover crop in crop rotations, making alfalfa highly suitable for sustainable agriculture (Shakirov *et al.*, 2012).

**Fig. 1.**
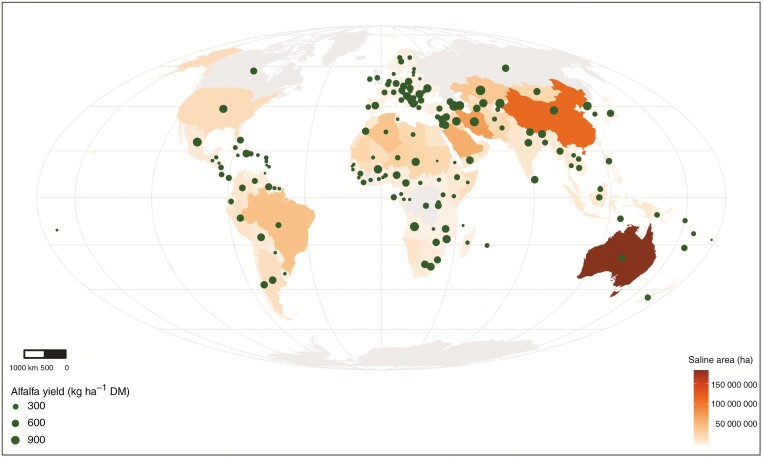
Global alfalfa production and salinization areas (with an ECe ≥ 4 dS m^−1^) displayed per country (based on Hassani *et al.*, 2020). Note: the grey areas located above 55° latitude (e.g. Canada, Russia and the UK) are not reported here. Alfalfa yield (kg ha^−1^ DM): the predicted increase of alfalfa’s highest occurring class yield from 2020 to 2080 in spatial units based on FAO-Climate Models (Fischer *et al.*, 2021).

In recent years, alfalfa has been popularized for cultivation in arid and semi-arid regions where soil salinization is a significant threat (Shi *et al.*, 2017; Al-Farsi *et al.*, 2020; Fischer *et al.*, 2021). Figure 1 shows the relation between total saline areas (ECe ≥ 4 dS m^−1^) and the predicted increase of maximum alfalfa yields over the next 60 years (from 2020 to 2080), according to FAO (Food and Agriculture Organization of the United Nations) data (Fischer *et al.*, 2021). This illustrates that a moderate increase in alfalfa production could be expected over the next decades, even in countries with large areas that are already severely salt-affected, such as Australia, China and Iran.

Salt stress affects plant development, growth and productivity mainly through two distinct processes, namely osmotic stress and ion toxicity (Munns and Tester, 2008; Yang and Guo, 2018; Zhao *et al.*, 2020; Singh *et al.*, 2022). Additional effects such as the over-production of reactive oxygen species (ROS) and a nutrient imbalance further increase the negative impact on the plant (Munns and Tester, 2008; Yang and Guo, 2018). Premature senescence and chlorosis of leaves, disturbed photosynthesis, phytohormonal imbalances, changes in root ion transport, energy and lipid metabolism, protein synthesis as well as molecular regulation at a genetic and genomic level can be induced by these stresses and are part of either the stress-induced pathological consequences or the plants’ adaptive strategy (Kaleem *et al.*, 2018). In contrast, knowledge of salt-tolerance mechanisms that allow alfalfa to maintain its normal physiological functions and growth with elevated levels of salinity is still limited, especially with regard to the large variation in salt tolerance among alfalfa varieties (Zhang and Wang, 2015). This review summarizes the recent progress and challenges of unravelling the mechanisms linked to salt stress in many different plants, with an emphasis on alfalfa, focusing on the interrelation among morphological changes, physiological and molecular mechanisms, and the potential of exploiting this knowledge towards improving salt tolerance of alfalfa and other industrially relevant crops.

## HOW DOES SALT STRESS AFFECT PLANTS, SPECIFICALLY ALFALFA?

The salt tolerance of a crop is often described by the soil salinity (ECe) threshold level for initial yield loss and relative yield reduction per unit increase in salinity above this threshold (Tanji and Kielen, 2002). With a threshold ECe for yield loss (calculated as shoot dry mass) at 2 dS m^−1^ (~22 mM NaCl) and a 7.3 % yield reduction per dS m^−1^, alfalfa is classified as moderately tolerant to salinity based on early reports using the threshold–slope linear model. This model is a response function with two line segments, a salinity threshold and a declining slope, describing how relative crop yield declines along a series of incrementally increasing root-zone salinity (Maas and Hoffman, 1977; Steppuhn *et al.*, 2005; Maas and Grattan, 2015). However, using a ‘threshold slope’ may not accurately describe crops’ actual responses to salinity and therefore might not be the most suitable approach to rate crop salt tolerance (Steppuhn *et al.*, 2005). As the importance of alfalfa in saline land management is rising, numerous alfalfa breeding programmes have been implemented to identify and select more salt-tolerant varieties. More recent studies, for example by Anderson *et al.* (2023) and Ashrafi *et al.* (2014), found that certain modern alfalfa varieties bred for salt tolerance can still achieve economic yields at soil salinities of 10–15 dS m^−1^ ECe over a 3–4-year period, exemplifying how current research tends to assess crop yield reduction more continuously instead of starting at a specific threshold point (Food and Agriculture Organization of the United Nations, 2022). Unfortunately, when measuring salinity tolerance, studies often deploy different measures such as shoot dry weight, total dry matter or grain yield, making it challenging to combine the individual studies into a generalized salinity tolerance ranking of crops. Based on the aforementioned measures, alfalfa can generally be classified as a salt-tolerant crop under field conditions with the potential tolerance of soil salinities exceeding 6.0–8.0 dS m^−1^ ECe, making it more salt-tolerant than other major crops such as rice (*Oryza sativa*), wheat (*Triticum aestivum*), durum wheat (*Triticum turgidum* subsp. *durum*), barley (*Hordeum vulgare*), soybean (*Glycine max*) and common bean (*Phaseolus vulgaris*; Table 1).

**Table 1.** Inter- and intraspecies variation of relative salinity tolerance among different plant species

Relative salt tolerance ranking	Criteria used to calculate ranking
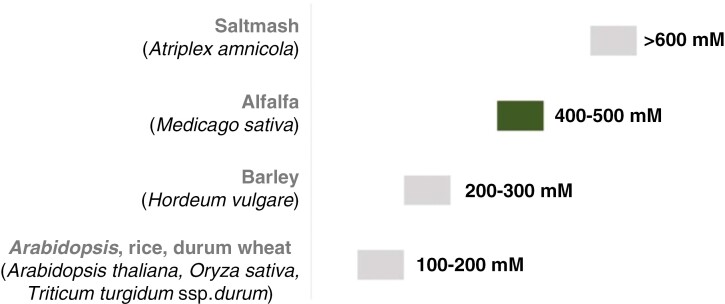	Based on the NaCl concentration of 100 % reduction in shoot dry weight (DW) relative to the DW in the absence of NaCl (Munns and Tester, 2008; Urbanavičiūtė *et al.*, 2021)
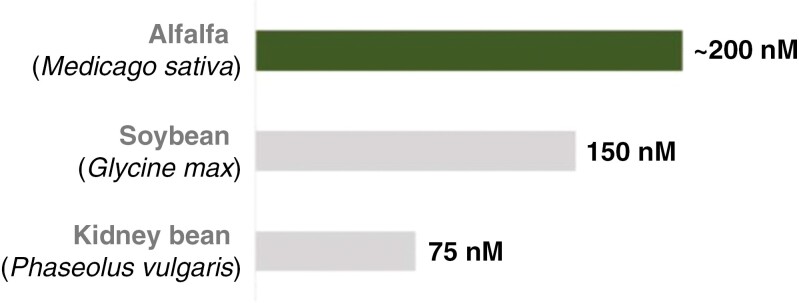	Based on the NaCl concentration at which a 50 % reduction of total dry matter (DM) is observed (Lawson *et al.*, 2009)
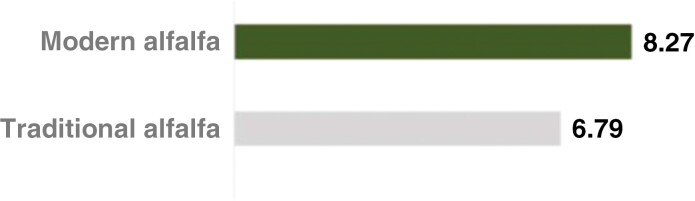	Based on the salinity tolerance index (STI; a non-linear model), equal to the salinity level at which 50 % of the non-saline crop shoot DW (C_50_) is achieved (Steppuhn *et al.*, 2005).

Below the species level, individual alfalfa varieties exhibit significant variability in their tolerance, surpassing that of many other crops (Zhang and Wang, 2015; Tani *et al.*, 2018; S. Fan *et al.*, 2023*a*), which can be utilized as a starting point for discussing the numerous mechanisms underlying salt tolerance. Leading up to this, we next describe the effect that salt stress has on above- and below-ground parts, first in crops in general and then in alfalfa specifically.

### Effect on the roots

The root system, which is the first organ sensing salinity, is responsible for salt uptake and transport to the above-ground parts of the plant (Koevoets *et al.*, 2016). Reshaping the root system architecture (RSA) is a common trait in plants facing edaphic abiotic stresses including salinization (Zou *et al.*, 2022). Typical examples of RSA remodelling strategies to cope with salinity include decreasing primary root length, number and length of both lateral roots and root hairs, surface areas, and average root diameter and volume, with the goal of limiting salt uptake at the cost of reduced water and nutrient acquisition (Koevoets *et al.*, 2016; Munns *et al.*, 2020; Karlova *et al.*, 2021; Zou *et al.*, 2022). Anatomical changes observed in rice, wheat and barley include shrinkage of the pericycle, exfoliation of root epidermal cells, and the disappearance of nucleoli and vacuoles, reducing root growth and cell viability (Liu *et al.*, 2019; Zeeshan *et al.*, 2020).

Salt-induced root K^+^ efflux has been widely reported for many plant species including *Arabidopsis*, barley and wheat, and can be considered as a metabolic switch for redirecting energy to defence reactions (Chen *et al.*, 2005; Jayakannan *et al.*, 2013; Rubio *et al.*, 2020). However, excessive K^+^ efflux-induced low cytosolic K^+^ concentrations can also lead to programmed cell death in the root cortex (Rubio *et al.*, 2020), resulting in aerenchyma formation as an undesired root trait in metabolically active root zones, which consequently inhibits shoot biomass accumulation (Guo *et al.*, 2015; Koevoets *et al.*, 2016; Escamez *et al.*, 2020).

**Fig. 2.**
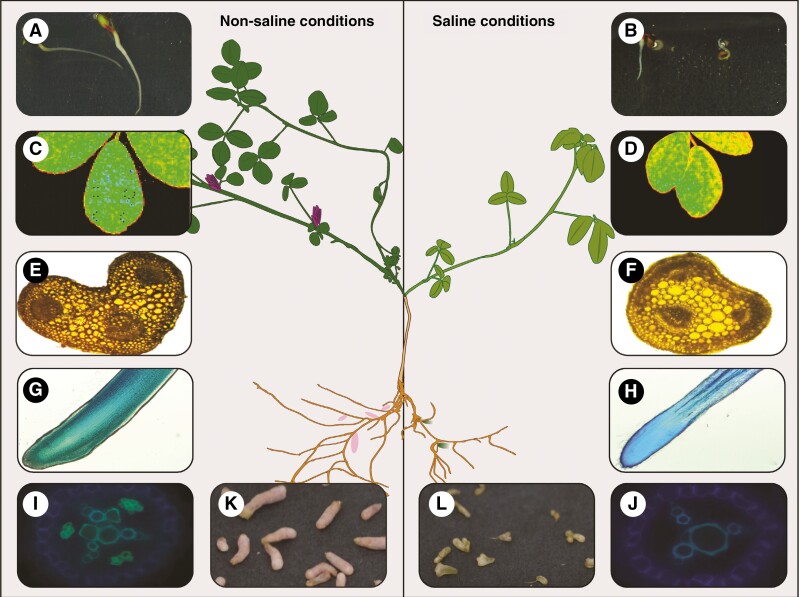
Comparison of alfalfa growing under non-saline and saline (150 mM NaCl) conditions. Typical seed germination after 3 d (A), while the process is drastically delayed under salt stress (B). Leaf morphology and maximal (Fm) chlorophyll fluorescence distribution for alfalfa grown in non-saline (C) conditions, while leaf area and Fm are decreased under saline conditions (D). Slightly decreased diameter and vascular bundles of salt-treated seedlings stems (F) compared to control conditions (E). Primary root tip (G, H) and cross-section (I, J) of alfalfa seedlings grown on agar plates, demonstrating a smaller diameter, a damaged meristem zone and root cap, as well as a decreased total number and area of xylem vessel area in salt-treated plants. A colour change from pink (K) to green (L) as well as a decreased nodule number and size can be observed for alfalfa grown in saline conditions.

Despite the importance of rootage for salt tolerance, in alfalfa, the morphological and physiological responses and the variability of the root system have been rarely assessed. Root growth in alfalfa appears to be less salt-arrested than shoot growth, and modern alfalfa varieties have developed deep and robust root systems that belong to one of the four types, depending on the alfalfa variety: (1) the tap-root type, (2) the branch-root type, (3) the creeping-root type and (4) the rhizomatous type, but most studies focus only on tap-rooted and branch-rooted varieties (Bucciarelli *et al.*, 2021; A *et al.*, 2022). Branch-rooted alfalfa varieties can be distinguished from tap-rooted varieties based on having longer and more abundant secondary and tertiary roots, without having an obvious taproot (Bucciarelli *et al.*, 2021). Branched and highly fibrous roots are probably advantageous for a higher alfalfa biomass yield and have been considered to play potential roles in the salinity avoidance mechanism in alfalfa (Vaughan *et al.*, 2002). Some alfalfa varieties can respond to environmental stresses by modifying the total stele and xylem vessel area as well as the size of the root system (Fig. 2G–J; Song *et al.*, 2019; Yu *et al.*, 2021). More specifically, alfalfa varieties with comparatively small root systems can grow a relatively large xylem vessel area (Fig. 2I, J) to maintain axial transport of water and nutrients during suboptimal conditions (Pan *et al.*, 2023). Under salt stress, many tap-rooted alfalfa varieties can maintain tap root growth while only showing a reduction in the number and length of lateral roots (e.g. secondary roots), leading to diminished salt uptake and improved energy conservation (Chinnaswamy *et al.*, 2018; Ansari *et al.*, 2019; Munns *et al.*, 2020; Yu *et al.*, 2021). However, whether and how those root trait modifications are regulated and if they are a result of active or adaptive plasticity under salt stress remains to be determined.

Besides changes in morphology and structure, physiological adaptations in the root also play a critical role in withstanding salt stress. While high soil salinity leads to a passive influx of Na^+^ ions into the root cells via the plasma membrane, a rapid K^+^ leakage from the cells is initiated within the first hour of salt stress imposition for many plant species (Jayakannan *et al.*, 2013). In multiple crops such as barley and wheat, the NaCl-induced K^+^ ‘leakage’ is stronger at the root apex than at the mature root zone, while in alfalfa leakage seems to be similar across different root zones (Chen *et al.*, 2005; Shabala and Cuin, 2008; Smethurst *et al.*, 2008; Zhang *et al.*, 2015; Rubio *et al.*, 2020). Under similar experimental conditions, the K^+^ efflux rate in the alfalfa root elongation zone appears to be much lower than for example in rice seedlings (Zhang *et al.*, 2015; Liu *et al.*, 2019). Transient K^+^ efflux rates ranging from ~200 to ~900 nmol m^−2^ s^−1^ are induced at the onset of NaCl stress in alfalfa roots, with the more salt-tolerant varieties having a superior K^+^ retention ability than salt-sensitive varieties (Smethurst *et al.*, 2008; Zhang *et al.*, 2015). Since a similar pattern has been observed also in other species, a low NaCl-induced K^+^ efflux from the roots could be seen as a reliable selection criterion for salt-resistant plant varieties (Smethurst *et al.*, 2008; Guo *et al.*, 2015; Xiong *et al.*, 2018).

### Effect on the root-associated microbiome

Salinity affects not only the plant itself, but also the highly diverse root-associated microbial communities (Qin *et al.*, 2016). Different plants harbour distinct microbial communities, which are usually dominated by plant-growth-promoting rhizobacteria (PGPR) and symbiotic endophytic bacteria (Vacheron *et al.*, 2013; Qin *et al.*, 2016). Under salt stress, plants can, sometimes rapidly, tailor their bacterial community structure to their needs by, for example, enriching halophilic bacteria, as demonstrated in peanuts (*Arachis hypogaea*) and glasswort (*Salicornia europaea*) (Qin *et al.*, 2016; Szymańska *et al.*, 2018; Xu *et al.*, 2020). Unfortunately, increased salinity often still leads to a reduced rhizosphere microbial diversity, lower microbial biomass, and ineffective symbiosis formation, as also seen in alfalfa (Xu *et al.*, 2020; W. Fan *et al.*, 2023*b*).

Alfalfa and other legumes do have an additional problem with salt stress because of its inhibitory impact on root nodule symbiosis. Depending on the host species, associations with rhizobia can be established within two different ‘types’ of nodules: determinate nodules (DNs) have a meristem at the periphery and lose meristematic activity shortly after initiation, resulting in a spherical shape (found in soybean and common bean), while indeterminate nodules (INs) possess a persistent meristem at their apex and elongate in a cylindrical shape such as in alfalfa, peas (*Pisum sativum*) and clover (*Trifolium* ssp.; Green *et al.*, 2019; Liu *et al.*, 2020). Following establishment, rhizobia differentiate into bacteroids, which can fix atmospheric nitrogen (N_2_) via nitrogenase activity. Relative to roots and shoots, nodules are more vulnerable to saline environments, with their functionality being more affected than their growth (Manchanda and Garg, 2008; Chakraborty *et al.*, 2021; Chakraborty and Harris, 2022), whereby INs usually have a structural advantage, more flexibility and resilience than DNs, which could contribute to better stress tolerance (M. Li *et al.*, 2021*a*; Iqbal *et al.*, 2022; Berrabah *et al.*, 2024).

Interestingly, in alfalfa, different levels of salinity have contrasting effects on nodulation processes: low to moderate concentrations of salt (30–100 mM NaCl) can actually improve IN formation and leghaemoglobin (oxygen-carrying plant haemoglobin) activity, whereas only under severe salt stress (>100 mM) nodule formation (Fig. 2K, L) and activity are negatively affected, mainly via inhibited root growth, secondary root formation, a reduction of nitrogenase activity and a damaged nodular structure (Redondo *et al.*, 2012; Shakirov *et al.*, 2012; Chinnaswamy *et al.*, 2018; Hosseini-Nasr *et al.*, 2023; Wan *et al.*, 2023). A 60–70 % reduction in N_2_ fixation can be observed in inoculated alfalfa at 200 mM NaCl, while other legumes such as common beans display the same decline already at 25 mM NaCl, with decreased nitrogenase activity being the most restrictive factor for N_2_ fixation (Faghire *et al.*, 2011; Palma *et al.*, 2013). Moreover, salt stress also accelerates nodule senescence, indicated by a colour change from pink to green (Fig. 2K, L; Redondo *et al.*, 2012). The senescence patterns of INs drastically diverge from those of DNs: in DNs, the occluded intercellular spaces and cell collapse starting from the centre of the nodules and extend radically (Verdoy *et al.*, 2004), while in contrast, structural alterations of alfalfa INs first appear in young, distal tissues (Redondo *et al.*, 2012).

### Effect on the stem

As the connecting organ between roots and leaves, the stem typically fulfils central functions for water and nutrient transport and storage while providing mechanical support for other above-ground organs. However, under salt stress, both stem height and diameter are generally reduced. Anatomically, the epidermis, phloem and xylem thickness, pith diameter, metaxylem diameter, as well as the number, length and diameter of vascular bundles are decreased, while excessive lignification of cells and disruption of xylem structure occur (Nassar *et al.*, 2020; Silva *et al.*, 2021). A decreased hydraulic conductivity in both the stem xylem and phloem can indirectly or directly be induced to restrict water flow including dissolved salts to the leaves (Nassar *et al.*, 2020), which is an important trait for salt tolerance via salt ion exclusion (see ‘Salt exclusion strategies’ below) across the plant kingdom, as seen in black gram (*Vigna mungo*), bald cypress (*Taxodium distichum*), coastal *Arabidopsis* ecotypes and more salt-tolerant potato (*Solanum tuberosum*) varieties (Jaarsma *et al.*, 2013; H. Li *et al.*, 2023*a*). Conversely, a significant increase of stem hydraulic conductance under mild salinity levels was found in moderately salt-tolerant tomatoes (*Solanum lycopersicum* L. cv. Naomi) when accumulated Na^+^ acts as osmoticum, beneficially raising the salt concentration in the xylem sap via xylem–phloem exchange (Trifilo *et al.*, 2013; Ketehouli *et al.*, 2019).

In alfalfa, the maintenance of stem growth is essential for generating biomass and especially for biofuel production, as the stem contains substantial amounts of cellulose and hemicellulose (Hojilla-Evangelista *et al.*, 2017). However, alfalfa stem (Fig. 2E, F) and petiole growth are more salt-affected than root or leaf growth (Nja *et al.*, 2018; Bhattarai *et al.*, 2020). Various salt-induced modifications with the aim of decreasing hydraulic conductivity, increasing cavitation resistance from salinity and mechanical tissue reinforcement can be observed in alfalfa, especially in the lower internodes close to the root (Nja *et al.*, 2018; Bhattarai *et al.*, 2020). Typical modifications include an altered differentiation of the cambium and its derivative tissues, increased cell wall thickening, lignification of phloem and xylem fibres, and damaged companion cells in the phloem complex (Nja *et al.*, 2018; Bhattarai *et al.*, 2020). Even though these modifications can be temporarily beneficial by, for example, reducing Na^+^ transport to the leaves, under continuous salt stress they inevitably lead to a decreased biomass and digestibility (Nja *et al.*, 2018; Bhattarai *et al.*, 2020).

### Effect on leaves

A widespread, general effect of salt stress on the foliage is an obvious colour change towards light-green or even yellow with an increased leaf thickness due to salt-induced expansion of leaf palisade mesophyll cells, while leaf length, width, number and surface area decrease (El-Hendawy *et al.*, 2005; Acosta-Motos *et al.*, 2017). Physiologically, the functionality of the photosynthetic apparatus can be perturbed, leading to significantly reduced stomatal conductance, transpiration and net photosynthetic rate, as has been shown for multiple plants including rice, barley and rosemary (*Rosmarinus officinalis*; Munns and Tester, 2008; Acosta-Motos *et al.*, 2017). Furthermore, stomatal closure decreases the transpiration rate to help maintain leaf cell turgor, but this also comes at the cost of lowering the photosynthetic rate (Kalaji *et al.*, 2011; Cabot *et al.*, 2014; Shi-chu *et al.*, 2019). Photoinhibition, mainly of photosystem (PS) II, and a reduction of the photosynthetic pigments chlorophyll (Chl) *a* and *b* as well as carotenoids limit photosynthesis even further (Adams *et al.*, 2013; Latrach *et al.*, 2014; Parihar *et al.*, 2015; Shi-chu *et al.*, 2019).

For alfalfa farmers, leaf growth inhibition is especially problematic given that more than 70 % of protein production depends on leaf biomass (Hojilla-Evangelista *et al.*, 2017; Guiza *et al.*, 2022). Numerous studies report a notably decreased leaf area (Fig. 2C, D) in many alfalfa varieties under salt stress, with less reduction observed in the more salt-tolerant varieties, which has been used as an argument for using the relative reduction of leaf area as a proxy for salt tolerance (Chinnaswamy *et al.*, 2018; Tian *et al.*, 2020; El Moukhtari *et al.*, 2021). Others argue that leaf area is only impacted under severe salt stress, while under moderate salt stress a young leaf, once initiated, will still be fully expanded, making leaf area an imprecise proxy for salt tolerance in alfalfa (Anower *et al.*, 2013; Guiza *et al.*, 2022). Besides, the photosynthetic capacity of alfalfa under salt stress is also restricted by non-stomatal factors such as the downregulation of dark reaction-related enzymes or photosystem damage rather than by stomatal limitations (Shi-chu *et al.*, 2019). Inhibition of PSII photochemical activity (Fig. 2C, D) results from blockages in the electron transport chain. Salt stress inhibits oxygen evolution complex (OEC) activity on the PSII donor side and inactivates electron transport to the secondary quinone (QB) on the PSII acceptor side, with the PSII acceptor side of alfalfa having a greater salt sensitivity than that of the donor side. Whereas photoinhibition usually happens predominantly in PSII, in alfalfa PSI is more salt affected and therefore more likely to experience photoinhibition (Shi-chu *et al.*, 2019). Thus, improving PSI activity or PSII photochemical efficiency, for example by increasing the activities of related enzymes (such as phospho-enol-pyruvate carboxylase), could lead to enhanced alfalfa photosynthetic capacity despite saline conditions.

### Effect on seeds

In most plants, salt stress will extend germination time or completely inhibit seed germination by causing electrolyte leakage and retarded development of storage vacuoles and amyloplasts (Ibrahim, 2016). The mobilization of storage reserves can be delayed by altering the form of residual protein bodies as well as the quantity of storage proteins and starch grains (Baranova *et al.*, 2011). Moreover, the translation initiation factors of salt stress-related proteins involved in seed germination can be affected as well (Ma *et al.*, 2017).

Interestingly, alfalfa can produce heteromorphic seeds with distinct colours, namely brown, yellow and green, which is a common phenomenon observed in halophytic (= able to grow under highly saline conditions) species such as *Suaeda salsa* (Liu *et al.*, 2018). These seeds display a different germination behaviour (Fig. 2A, B) under salt stress; for example, lighter-coloured, yellow and green alfalfa seeds exhibit better germination characteristics than dark brown seeds (Liu *et al.*, 2018; Xie *et al.*, 2023). This trait might be linked to the corresponding phytohormone content in the seeds of diverse colours, with especially the abscisic acid (ABA) content being higher than the growth-promoting hormones indoleacetic acid (IAA) and gibberellins (GA) in brown seeds (Xie *et al.*, 2023).

## HOW DO DIFFERENT CROPS COPE WITH SALT STRESS?

### The extremes: salt exclusion vs. salt inclusion strategies

**Fig. 3.**
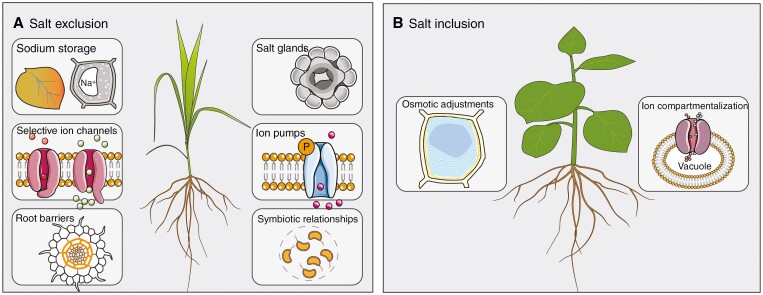
Contrasting strategies to deal with excess salt. The salt exclusion strategy (A) employs different mechanisms than the salt inclusion strategy (B); few plants use a combination of both or can switch between strategies.

Plants have evolved various mechanisms to cope with salinity stress. Those mechanisms can be broadly divided into salt exclusion (Fig. 3A) and salt inclusion strategies (Fig. 3B). Salt exclusion strategies at the whole-plant level aim to avoid toxic salt ion accumulation in leafy shoots, especially new source leaves, for maintaining photosynthesis, transpiration and growth, whereas salt inclusion strategies involve mechanisms to tolerate and manage high salt concentrations within the plant (Acosta-Motos *et al.*, 2017; Chen *et al.*, 2018; Ketehouli *et al.*, 2019). Most monocot glycophytes such as maize (*Zea mays*), wheat and rice are salt excluders with a dramatically lower salt tolerance than halophytes (Chen *et al.*, 2018), whereas dicot glycophytes (including soybean) and halophytes, such as *Plantago coronopus*, *Cakile maritima* and *Leptochloa fusca*, normally are salt includers (Ledesma *et al.*, 2016; Hmidi *et al.*, 2018). However, some halophyte grasses such as common reed (*Phragmites australis*) also follow salt exclusion strategies (Achenbach *et al.*, 2013). Of course, these two types of behaviour are extreme, and many species can incorporate behaviours characteristic of both types (Acosta-Motos *et al.*, 2017).

#### Salt exclusion strategies

1. Salt exclusion at the root:

Some plants with salt-exclusion strategies aim to prevent salt from entering their root cells. They achieve this by various mechanisms, including:

- Restricting Na^+^ influx: Plants can restrict salt ions uptake via, for example, downregulation of the voltage-dependent non-selective cation channel (NSCC), some high-affinity K^+^ transporters (HKTs) and K^+^ channels; in addition, for some channels/transporters such as the Shaker-type K^+^ channel in *Arabidopsis* (AKT1) and HKTs in rice (OsHKT2; Table 2), it has been argued that they could exhibit a higher selectivity for K^+^ than for Na^+^ (Assaha *et al.*, 2017; Yang and Guo, 2018).
- Ion pumps for Na^+^ efflux: Energy-dependent plasma membrane proton pumps establish an electrochemical gradient of protons to energize the transport of solutes and metabolites across the plasma membrane, such as the Na^+^ transport out of the cells and K^+^ absorption (Silva *et al.*, 2010; Chen *et al.*, 2018; Ketehouli *et al.*, 2019).
- Root barrier formation: Some plants develop barriers in their roots by deposition of suberin and/or lignin that block the passage of salts, preventing their entry into the vascular system (Krishnamurthy *et al.*, 2014).

**Table 2.** Salt-responsive genes and enzymes related to signalling perception and transduction pathways

Gene / Enzyme	Description	Function under salt stress
*MsMYB4*	Alfalfa R2R3-MYB transcription factor	Directly involved in salt tolerance in an ABA-dependent manner (Dong *et al.*, 2018)
*MsWRKY33*	Transcription factor belonging to the WRKY superfamily in alfalfa	Regulating the responses to biotic and abiotic stress in plants
OsHKT2;1/2	A hybrid of OsHKT2;1 (Na^+^-selective) and OsHKT2;2 (K^+^-selective) in rice	Mediating K^+^ and Na^+^ uptake (Assaha *et al.*, 2017)
SIMK	Stress-induced mitogen-activated protein kinase	Salt-responsive MAPK affecting root hair growth, early nodulation, petiole and leaf development (Hrbáčková *et al.*, 2021)
SIMKK	Dual-specificity protein kinase	Negative regulator of salt-stress response during the alfalfa seedling stage but not during seed germination (Hrbáčková *et al.*, 2021)

1. Salt exclusion at the shoot:

Specific mechanisms limit salt ion transport to younger source leaves and other aerial photosynthetic tissues:

- Sodium compartmentalization: By transporting excess Na^+^ to older leaves or specialized root and stem cells, plants can keep the sodium away from essential metabolic processes in actively growing, young tissues (Wang *et al.*, 2012; Mansour and Salama, 2019).
- Salt glands/bladders: Certain species of plants, especially halophytes, have salt glands or bladders on their leaves that excrete excess salts, maintaining a lower salt concentration in leaf tissues (Kiani-Pouya *et al.*, 2019; Zhao *et al.*, 2023).

1. Salt exclusion through symbiosis:

Mutualistic root-associated microorganisms, both endo- and ectosymbiotic, can enhance plant tolerance to salinity, mainly via mediating Na^+^ homeostasis and nutrient uptake:

- Gene regulation: Some PGPR and arbuscular mycorrhiza can regulate the expression of ion transporter genes such as HKT1, SOS1 and NHX to not only limit Na^+^ entry into the roots but also facilitate shoot-to-root Na^+^ recirculation (Zhang *et al.*, 2008; Wang *et al.*, 2021).
- Biofilm formation: About one-third of halotolerant (= adapted to conditions of high salinity) rhizobacteria can form biofilms, acting as a protective barrier and restricting Na^+^ influx into the roots (Haque *et al.*, 2022).

#### Salt inclusion strategies

Some plants have developed two main mechanisms to tolerate high salt concentrations within their cells and organelles in different plant tissues while growing under saline conditions, known as tissue tolerance (Hmidi *et al.*, 2018; Ketehouli *et al.*, 2019):

- Ion compartmentalization: By sequestering Na^+^ into cell vacuoles, plants can minimize the harmful effects on essential cellular processes (Chen *et al.*, 2018; Ketehouli *et al.*, 2019; Mansour and Salama, 2019).
- Osmotic adjustment: Plants can accumulate compatible solutes (e.g. proline and glycine betaine) in the cytoplasmic compartments to balance the reduced solute potential in the vacuole, preventing water loss from cells (Mansour and Salama, 2019).

### Crosstalk between salt exclusion and inclusion strategies to prevent ion toxicity

Plants have evolved to assimilate nutrients from low ionic environments but are often intolerant to high Na^+^ concentrations, which results in Na^+^ toxicity being one of the primary mechanisms of cell damage in most plants under salt stress. K^+^, on the other hand, is essential to maintain electrolyte and osmotic balance in the cells (Kader *et al.*, 2006). A Na^+^/K^+^ ratio optimal for growth and development is usually 1–10 mM Na^+^/100–200 mM K^+^ in the cytosol of plant cells (Kader *et al.*, 2006). Therefore, it is essential that the cytosol can exclude Na^+^ to lower the concentration or maintain a low Na^+^/K^+^ ratio under salt stress. Salt-resilient plants, especially halophytes, are able to flexibly maintain ion/water movement across plant compartments and utilize inorganic ions to minimize the energetic cost of salt tolerance (Perri *et al.*, 2019).

Alfalfa deploys multiple, tissue-specific strategies to optimize the Na^+^/K^+^ ratio under salt stress. For example, some alfalfa varieties build up salt resilience in the stem by converting restricted Na^+^ transport to aerial tissue under moderate salinity (around 80 mM NaCl) to high salt ion trapping in stems and leaves without significantly harming the plants (Bhattarai *et al.*, 2021). In these relatively salt-tolerant alfalfa varieties, the mobilization and partitioning of carbohydrates and lipids is still active in coupled xylem and phloem, which is in contrast to more salt-sensitive varieties where this only occurs in the xylem. This exemplifies how some alfalfa varieties can switch between salt exclusion and inclusion strategies under different salinity conditions. More specifically, varieties with a higher salinity tolerance better manage to retain Na^+^ in their roots, recycle K^+^ between the shoots and roots to prevent ionic toxicity, and re-establish ion homeostasis under moderate (around 80 mM) NaCl stress (Smethurst *et al.*, 2008; Xiong *et al.*, 2018). Under severe salinity stress (around 120 mM NaCl), alfalfa can translocate Na^+^ over long distances to the shoots and highly compartmentalize Na^+^ ions into leaf cell vacuoles, where they serve as an energy-cost-efficient osmoticum for turgor maintenance (Bhattarai *et al.*, 2021). Moreover, the Salt Overly Sensitive (SOS) signalling pathway, comprising SOS1, SOS2 and SOS3, is essential for Na^+^ exclusion (Luo *et al.*, 2019). In alfalfa roots, intra- and intercellular Na^+^ detoxification mechanisms are supported by differential expression of *SOS*-related genes and vacuolar Na^+^/H^+^ antiporters (NHXs), whereas in the leaves *NHX*s are mainly deployed for Na^+^ inclusion (Lei *et al.*, 2018; Luo *et al.*, 2019). Based on this, promising efforts in genetic modification have been made to maintain a low Na^+^/K^+^ ratio in alfalfa under salt stress, for example via heterologous expression of *SsNHX1* and *ZxNHX* (Table 2) from either the halophytic *Salsola soda* or *Zygophyllum xanthoxylum*, which increased resistance of alfalfa to severe salinity stress between 200 and 400 mM NaCl (Li *et al.*, 2011). Also, the heterologous expression of a vacuolar H^+^-PPase (*ZxVP1-1* from *Zygophyllum xanthoxylum* or *ScVP* from *Suaeda corniculate*) as well as overexpression of the *AVP1* and *SOS* genes improved the performance of alfalfa at 200–300 mM NaCl (Long *et al.*, 2015; Bao *et al.*, 2016). In addition, heterologous expression of alfalfa *MsNHX1* and *MsRCI2s* (Table 2) have shown great potential for conferring salt tolerance in *Arabidopsis* even at relatively high salt concentrations of 175–200 mM NaCl (Bao-Yan *et al.*, 2008; Wang *et al.*, 2019). These examples demonstrate the potential of fine-tuning pathways and mechanisms involved in maintaining a low Na^+^/K^+^ ratio to improve salt tolerance in alfalfa.

### Osmotic stress signalling and regulatory pathways involved in salt acclimation

The increased concentration of salt in plant tissues growing on salinized soils is creating osmotic stress. Several signalling pathways are directly activated by increased osmotic stress, for example calcium-dependent protein kinases, calcineurin B-like protein kinases, mitogen-activated protein kinases (MAPKs) and pyrabactin resistance-likes-2C protein phosphatase-SNF1-related protein kinase 2s (PYL-PP2C-SnRK2s), which are also well-characterized in alfalfa (Luo *et al.*, 2019). Many prospective candidates for genetically improving tissue tolerance have emerged from these signalling perception and transduction pathways. In alfalfa, stress-induced mitogen-activated protein kinase (SIMK) has been identified as an alfalfa salt-responsive MAPK. SIMK and its upstream dual-specificity protein kinase (SIMKK; Table 2) predominantly co-localize in the nucleus in root epidermal cells. Under salt stress, they relocate into cytoplasmic compartments and the growing root hair tips (Ovečka *et al.*, 2014; Hrbáčková *et al.*, 2021). SIMK and SIMKK–SIMK modules (but not sole SIMKK) can promote alfalfa root hair growth and early nodulation as well as petiole and leaf development (Hrbáčková *et al.*, 2021). Moreover, V-myb avian myeloblastosis viral oncogene homologue (MYB) transcription factor (TF) families are biologically important components for the regulation of osmotic stress-induced signalling pathways and osmotic tolerance (Ho-Plágaro *et al.*, 2019; Luo *et al.*, 2019). In particular, R2R3-MYB TF genes such as *MsMYB4* (Table 2) have been shown to directly increase alfalfa’s ability to tolerate salinity in an ABA-dependent manner (Dong *et al.*, 2018). Furthermore, overexpression of the salt-responsive TF *MsWRKY33* (Table 2) improved the salt tolerance of alfalfa at 200 mM NaCl via interaction with the functional fragment *MsCaMBP25*, probably blocking Ca^2+^ signal transduction caused by salt stress (Ma *et al.*, 2023).

Nevertheless, as recently reviewed by Moukhthari *et al.* (2024), further research is needed to identify more TFs and signalling pathways that contribute to salt acclimation in legumes and alfalfa in particular.

### Osmotic adjustment and osmoprotectants for tissue tolerance

The accumulation of osmoprotectants is one of the most prevalent salt-inclusion strategies for promoting osmotic balance and maintaining cell turgor in plant growth under salt stress (Singh *et al.*, 2022). Proline is the most common osmolyte used in various organisms from bacteria to plants and can contribute to both ion homeostasis and antioxidant defence for plant adaptation to salinity (de Freitas *et al.*, 2018; Singh *et al.*, 2022). It is worth noting that the direct correlation between proline accumulation and improved salt tolerance in alfalfa is still of debate. Proline accumulation has been argued to be a reaction to salt stress rather than a response associated with salt tolerance in alfalfa varieties (Campanelli *et al.*, 2013). Recently, the affinity of proline with polyamines and γ-aminobutyric acid (GABA) as well as the roles of polyamines and GABA in symbiotic alfalfa under salt stress have attracted increased attention. All three compounds share glutamate as their common precursor, and the upregulation of polyamine metabolism seems to stimulate proline and GABA accumulation under salt stress (Legocka *et al.*, 2017). Although proline is more prevalent than polyamines in the initial response of noduled alfalfa to salinity, polyamines such as homospermidine and cadaverine can highly accumulate under long-term salt stress. These polyamines not only share similar functions with proline but also cross-talk with phytohormones such as brassinosteroids (BRs) to assist the establishment of rhizobia symbioses (López-Gómez *et al.*, 2014, 2017). Meanwhile, GABA is especially abundant in salt-stressed seedlings and nodules, where it might function as a signalling molecule for alfalfa–rhizobia communication and as energy source for bacteroids (Bertrand *et al.*, 2016). GABA can also participate in the amino acid cycle between bacteroids and the cytoplasm, assisted by branched-chain amino acid transporters across the peribacteroid membrane in nodules (Bertrand *et al.*, 2016). However, not only individual but also total amino acid production and composition are associated with the osmo-adaptation and salt tolerance in (nodular) alfalfa (Bertrand *et al.*, 2016; Mansour and Ali, 2017). Specifically, accumulation of glutamate-family amino acids (asparagine, proline, GABA, glutamine, glutamate and ornithine) is generally associated with osmo-adaptation, ammonium assimilation and energy conservation under salinity stress, while the accumulation of aspartate-derived amino acids such as lysine and methionine reflects salt sensitivity (Bertrand *et al.*, 2016). Interestingly, the adjustment of amino acid composition seems to be salt-responsive in roots and leaves of alfalfa, while the composition in the nodules is mostly rhizobial-strain dependent (Bertrand *et al.*, 2016).

### Antioxidant defence system

The antioxidant system, depending on the tricarboxylic acid (TCA) cycle providing the reductant NADPH, plays an essential role in scavenging ROS and conferring tissue salt tolerance in plants (Naeem *et al.*, 2020). Its main constituents are the antioxidant enzymes superoxide dismutase (SOD), peroxidase, catalase (CAT) and ascorbate peroxidase, as well as non-enzymatic antioxidants mainly from glutathione (GSH) metabolism (Benabderrahim *et al.*, 2020; Li *et al.*, 2020; Naeem *et al.*, 2020). In recent years, an abundance of novel antioxidants mitigating salt stress have been uncovered in several species, including alfalfa, such as the nicotinamide adenine dinucleotide (NAD) synthetase, which is involved in ROS regulation and detoxification as a salt-responsive protein (Zilli *et al.*, 2008; Hashida *et al.*, 2010; Gao *et al.*, 2019). The NAD redox state affects the production of free radicals and governs ATP production in both the cytosol and mitochondria. Other relevant antioxidative participants include flavonoids and the numerous proteins involved in their biosynthesis, such as isoflavone reductases (IFRs; Table 3). Flavonoids can serve as potent antioxidants in plants, *inter alia* by chelating metal ions, affecting the lipid packing order of the membrane, inhibiting the superoxide-driven Fenton reaction and regulating the activity of alternative oxidases (Fini *et al.*, 2011; Samanta *et al.*, 2011). Structure–activity relationship assays have shown that the spatial arrangement and number of sugar chains are closely linked to the antioxidant properties of flavonoids: flavonoids with two sugar molecules attached generally show a stronger ability to trap free radicals than those with three sugars, which also holds true for novel antioxidative flavonoids recently extracted from alfalfa, including 6,8-dihydroxy-flavone-7-*O*-β-d-glucuronide and 6-methoxy-8-hydroxy-flavone-7-*O*-β-d-glucuronide (Liang *et al.*, 2011; Rafińska *et al.*, 2017). Lastly, haem oxygenase (HO) is a non-canonical cytoprotective enzyme against oxidative stress, in particular isozyme HO-1, whose antioxidative properties are well-studied in mammals but not yet in plants (Ryter and Tyrrell, 2000). The expression of HO is provoked when the activities of classical antioxidants are inhibited by salt stress in nodules of soybean to effectively support N_2_ fixation. Two independent studies report that alfalfa *MsHO1* provides significant benefits against oxidative stress, with a higher gene and protein expression in shoots and nodules than in roots (Zilli *et al.*, 2008; Fu *et al.*, 2011). In alfalfa, most genes related to oxidation/reduction are expressed tissue-specifically or with a higher expression in the roots, resulting in a more robust antioxidative system in the roots than in shoots or nodules under salt stress (Rahman *et al.*, 2015; Xiong *et al.*, 2017; Song *et al.*, 2019). For example, *CAT* gene expression is dramatically increased in roots after salt shock treatment but not in the nodules or leaves, which could be due to their different protective roles in signalling transduction metabolic channelling (Mhadhbi *et al.*, 2011*b*).

**Table 3.** Salt-responsive genes and enzymes related to antioxidant defence systems

Gene / Enzyme	Description	Function under salt stress
*CAT*	Catalase	Involved in the protection of signalling components for salt stress defence, especially related to H_2_O_2_ scavenging (Mhadhbi *et al.*, 2011*b*)
GalLDH	l-Galactono-1,4-lactone dehydrogenase	The high expression of GalLDH supports ascorbate biosynthesis in alfalfa nodules (Becana *et al.*, 2010)
IFR	Isoflavone reductase	A key enzyme for isoflavone biosynthesis; contributes to salt stress tolerance of alfalfa by, for example, regulating ROS homeostasis (Rahman *et al.*, 2015)
*MnSOD*	Manganese superoxide dismutase (SOD)	Manganese SOD is involved in antioxidation and redox regulation (Becana *et al.*, 2010)
*MsHO1*	Alfalfa haem oxygenase 1 gene	Potentially functions in oxidative responses; possible involvement in salt tolerance through nitric oxide signalling pathway (Fu *et al.*, 2011)
sodB-C	Encoding FeSOD and Cu/ZnSOD, respectively	Contributes to ROS detoxification (Queiroux *et al.*, 2012)
XDH and its isoforms	Xanthine dehydrogenase	Increases ROS scavenging by producing uric acid in nodules (Shakirov *et al.*, 2012)

Nodules of alfalfa have specific protective mechanisms to alleviate oxidative stress, which seems especially important giving that nodules are always at risk for ROS overproduction, even under normal conditions (López-Gómez *et al.*, 2014). In addition to structural alterations in the inner cortex and a diffusion barrier regulating oxygen diffusion, alfalfa nodules have a complex antioxidant system including abundant protective isozymes and molecules, for example SODs, galactonolactone dehydrogenase (GalLDH), xanthine dehydrogenase (XDH) and their isoforms (Table 3; Becana *et al.*, 2010; Mhadhbi *et al.*, 2011*a*; Queiroux *et al.*, 2012; Shakirov *et al.*, 2012). After it had been shown that this nodular ROS detoxification system can be altered and potentially strengthened by heterologous overexpression of flavodoxins (small electron-transfer proteins) in alfalfa-associated rhizobia (Redondo *et al.*, 2009), inducing nodulation with a modified redox capacity has become a promising avenue for improving nitrogen-fixation and general plant performance under salt stress in alfalfa and other nodule-forming crops (Coba de la Peña *et al.*, 2010; Redondo *et al.*, 2012).

### The role of specialized (secondary) metabolites for salt tolerance

While considerable attention has been paid to primary (general) metabolites such as sugars, amino acids and lipids for their functions in preventing osmotic and oxidative stress, an increasing body of literature indicates the importance of plant specialized metabolites (SMs) for salt tolerance (Benjamin *et al.*, 2019; Meng *et al.*, 2022; Rajkumari *et al.*, 2023). Plant SMs, mainly phenolics, terpenes and nitrogen-containing compounds (alkaloids, glucosinolates and cyanogenic glycosides), are not directly involved in primary growth and development, but can greatly increase the plants’ fitness and confer stress tolerance (Wu *et al.*, 2023). SM profiles are often very species-specific and even fluctuate within the same species when facing different environmental factors (Sarri *et al.*, 2021), as exemplified by the increased production of species-specific alkaloids using NaCl as an elicitor in black nightshade (*Solanum nigrum*) and castor beans (*Ricinus communis*; Ali *et al.*, 2007; Šutković *et al.*, 2011). Furthermore, terpenoids as the predominant constituents of essential oils are accumulating under high salinity in many mint (*Mintha*) species and common sage (*Salvia officinalis*) (Aziz *et al.*, 2008; Tounekti and Khemira, 2015).

**Fig. 4.**
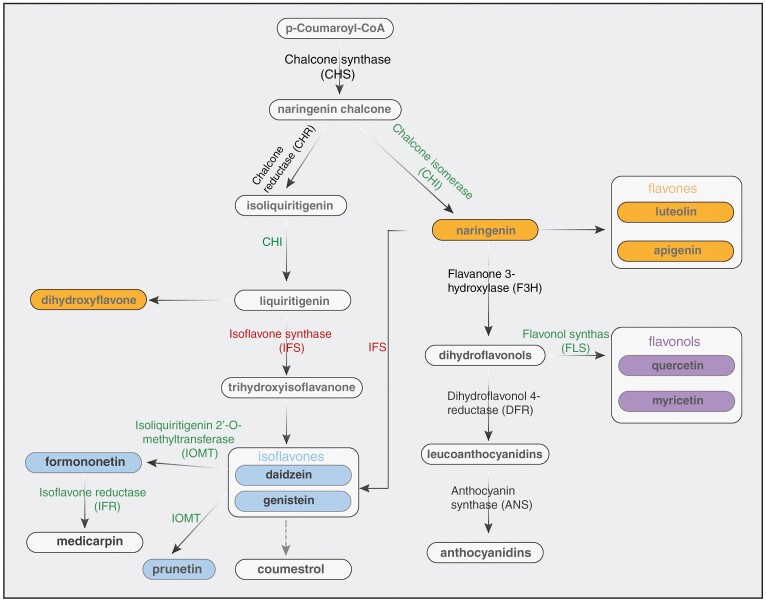
Up- and down-regulation of alfalfa flavonoid biosynthetic pathways under salt stress. Green: up-regulated; red: down-regulated; the expression of the other genes is not significantly altered by salt stress; grey-dotted line: pathway is still unclear (Xiong *et al.*, 2017; Liu *et al.*, 2021).

Alfalfa can equivalently alter its SM profile under salt stress (Sarri *et al.*, 2021). Most prominently, alfalfa possesses distinctive, fingerprint-like profiles of flavonoid compounds, which are one of the largest groups within the phenolics. Luteolin and apigenin (flavones) glycosides are abundant in aerial parts of alfalfa, while isoflavonoids, including formononetin, medicarpin, coumestrol and the genistein-derivative prunetin, are the predominant class of flavonoids in nodules and roots (Mishima *et al.*, 2015; Gifford *et al.*, 2018; Tao *et al.*, 2023). Under salt stress, genes involved in flavonoid biosynthesis, specifically CHS, CHI, FLS, DFR, IFR, CHR, ANS, F3H, IFS and IOMT (Fig. 4) are tightly regulated in coordination with the phytohormones jasmonic acid (JA), gibberelic acid and ABA, leading to increased levels of total and specific flavonoids such as dihydroquercetin and dihydromyricetin (Wang *et al.*, 2023). In addition to their antioxidative properties discussed in the previous section, one of the main benefits of flavonoids in alfalfa is their strengthening effect on the symbiotic communication with rhizobia. This can be important for salinity stress, because for certain legumes increased salt tolerance has been measured after inoculation with specific rhizobia (Qu *et al.*, 2016). Flavonoids can activate the transcription of bacterial *nod* genes (Table 4), which is a prerequisite for nodule formation (Phillips *et al.*, 1994; Sugiyama and Yazaki, 2014). The accumulated flavonoids, particularly isoflavonoids, could potentially assist in preventing early symbiosis inhibition caused by salt stress in roots based on studies of multiple legumes, for example via controlling auxin transport to promote the localized auxin accumulation required for nodule development or by possibly alleviating the inhibitory effect of salinity on *nod* gene expression (Abd-Alla *et al.*, 2014; Dong and Song, 2020; Chakraborty *et al.*, 2021). Certain flavonoids (e.g. daidzein, luteolin-7-*O*-glucoside and quercetin-3-*O*-galactoside) furthermore contribute to the high species-specificity of the alfalfa–rhizobia symbiosis as chemoattractants and growth stimulants of, for example, *Ensifer* (formerly *Sinorhizobium*) *meliloti*, *Sinorhizobium medicae* and *Rhizobium mongolense* (Djedidi *et al.*, 2011; Weston and Mathesius, 2013).

**Table 4.** Salt-responsive genes and enzymes related to alfalfa–microbe associations

Gene / Enzyme	Description	Function under salt stress
*bet*	Glycine betaine synthase	Involved in betaine synthesis and transport in *Sinorhizobium meliloti* (Roumiantseva and Muntyan, 2015)
*nif*	Nitrogen fixation genes	Encodes enzymes involved in nitrogen fixation, for example nitrogenase (Chinnaswamy *et al.*, 2018)
*nod*	Bacterial nodulation genes	A key bacterial signal gene to induce nodulation (Chinnaswamy *et al.*, 2018)

In addition to flavonoids, alfalfa accumulates lignans and some unique saponins under salt stress, mainly medicagenic acid, zahnic acid tridesmoside, hederagenin, and soyasapogenol A and B (Sarri *et al.*, 2021; Wang *et al.*, 2023), which have been shown to act as photoprotectants, osmolyte production stimulators and ROS scavengers in alfalfa and soybean (Soliman *et al.*, 2020; Sarri *et al.*, 2021). Lastly, a pan-transcriptomic study on contrasting alfalfa germlines has revealed a high salt-responsiveness of a monoterpene synthase catalysing the formation of nerol, but the role of terpenoids for alfalfa salt adaptation and their regulation are still largely unknown (Medina *et al.*, 2021).

## DESIGNING NOVEL ALFALFA VARIETIES FOR SALT-PROOF AND SUSTAINABLE AGRICULTURE

Driven by the pace of rising salinity levels and fuelled by the genetic variability within and between alfalfa populations, multiple useful strategies have been proposed to realize sustainable alfalfa production. These strategies would also be advantageous in complementing empirical breeding programmes (e.g. yield-based approaches), and in further extending the knowledge to other crops.

### Reshaping the root system

Given the apparent key role of root plasticity and the emergence of extensive phenotyping platforms, designing roots for growth on saline lands serves as a promising strategy for cultivating alfalfa that can resist or avoid salinity without compromising yields (Uga, 2021; Bucciarelli *et al.*, 2021; P. Li *et al.*, 2021*b*). The first step in this direction could be to define a salt-proof alfalfa RSA ideotype, allowing for fast seedling establishment, reproducibility and stable crop production. Towards this step, we suggest a tighter integration of crop ecophysiology with functional genomic analyses, as per the following example. Specific surface area (SA) and specific root volume (RV), calculated as the ratios of SA or RV to the dry weight of roots, are positively correlated with salt tolerance in alfalfa by being involved in root hydraulic conductivity and proliferation (Yu *et al.*, 2021). SA and RV are physiologically regulated by the crosstalk between IAA, ABA, cytokinins, ethylene, JA and BRs (P. Li *et al.*, 2021*b*). Therefore, modification of critical genes involved in these phytohormone signalling pathways, such as the PIN-FORMED 2 (PIN2) auxin efflux carrier regulating the directional root growth away from salt, can help the salt-resistance oriented design of RSA.

### Enhancing K^+^ homeostasis

Besides anatomical and morphological aspects, the roots’ ability to retain K^+^ plays a critical role in plant salt tolerance (Guo *et al.*, 2008; Sun *et al.*, 2009). Several ion channels such as DA-KORCs, DA-NSCCs, the K^+^ channel blocker TEA and the AtCNGC10 channel (Table 5) have been genetically engineered to prevent NaCl-induced K^+^ efflux from plants (Guo *et al.*, 2008; Sun *et al.*, 2009). Detecting and editing the related channels towards maintaining K^+^ homeostasis would greatly improve salt tolerance and empower the current alfalfa breeding system.

**Table 5.** Salt-responsive genes and enzymes related to intra- and intercellular K^+^/Na^+^ homeostasis

Gene / Enzyme	Description	Function under salt stress
AtCNGC10	Cyclic nucleotide-gated channels in the plasma membrane transport K^+^ and other cations	Potential channel providing Na^+^ influx and K^+^ efflux at the root/soil interface under salt stress (Guo *et al.*, 2008)
DA-KORC	Depolarization-activated outward rectifying K^+^ channels	K^+^-permeable channel, conducting outward potassium flux (Demidchik and Maathuis, 2007)
DA-NSCC	Depolarization-activated non-selective cation channel	Catalyses passive fluxes of cations through membranes (Demidchik and Maathuis, 2007)
*NHX1* (*MsNHX1*, *SsNHX1*)	Vacuolar Na^+^/H^+^ antiporter	Vacuolar membrane sodium/proton exchangers, controling vacuolar pH and K^+^ homeostasis (Li *et al.*, 2011; Wang *et al.*, 2019)
*RCI2* (*MsRCI2*)	Rare cold-inducible 2-like gene family	Regulating cation flux and membrane potential (Long *et al.*, 2015).

### Mobilizing rhizobial and non-rhizobial bacteria

The benefits of alfalfa–rhizobia symbiosis have received extensive attention as being fundamental for designing stress-resilient roots and aiding sustainable agriculture, for instance by decreasing the necessary nitrogen input in farming systems (Stokstad, 2019). In addition to the aforementioned compounds for chemical communication, the polymorphism of *bet* genes (Table 4) in *S. meliloti* strains evolutionarily determines the symbiotic efficiency with host alfalfa ecotypes under various salty environments (Roumiantseva and Muntyan, 2015). In addition to rhizobia, non-rhizobial bacteria can also inhabit alfalfa nodules. Two examples are *Pantoea agglomerans*, a salt-resistant bacterium that can induce the development of nodule-like structures in the roots (Noori *et al.*, 2021), and *Bacillus megaterium* NMp082, containing *nifH* and *nodD* genes analogous to *S. meliloti* (Chinnaswamy *et al.*, 2018). Even though the role of non-rhizobial plant growth-promoting bacteria (PGPB) has not yet been fully unravelled, these endophytic non-rhizobial PGPB possess multiple beneficial traits and the interaction with rhizobia points to potential applications for improving nodulation and sustaining alfalfa growth under salt stress. Further extending this knowledge about microbial associations to other crops could be a sustainable way to improve crop performance on saline soils. This might, for example, be achieved via genetic engineering to grow indeterminate instead of determinate nodules or to ‘re-awaken’ plant–microbiota alliances in crops currently without nodulation, given that the ancestors of most modern crops were able to form nodules but lost this ability during recent evolution (Martin *et al.*, 2017).

### Fine-tuning the (specialized) metabolic fingerprint

The identification of specific metabolites and associated biosynthesis pathways has largely contributed to alfalfa-specific strategies for salt tolerance. Metabolic fingerprinting and profiling technologies, combined with transcriptomic approaches, are becoming a targeted and powerful tool for designing salt-resistant alfalfa. In addition to SMs, recent studies have identified salt-inducible lipidomic changes including remodelling of lipid profiles, storage lipid conversion and differential gene expression of lipid-related genes, which indicate potential for eliciting salt tolerance in alfalfa by tapping into the potential of lipids for membrane structure repair and signal transduction (Yuan *et al.*, 2022; M. Li *et al.*, 2023*b*). However, despite long-read sequencing and high-resolution mass spectrometry enabling more accurate gene, protein and metabolite identification and quantification, the large number and diversity of metabolites and their underlying pathways and mechanisms under salt stress still warrants further examination. Moreover, the causality between selected alfalfa varieties possessing the targeted traits and their high yield/quality on saline soils is still largely lacking. Integrated studies with long-term time series are urgently required to determine the critical characteristics of more resistant varieties. With this knowledge, targeted selection strategies could be designed to develop salt-tolerant varieties that are required to future-proof sustainable crop production on salinized soils.

## CONCLUSION

Numerous alfalfa varieties have developed salt-tolerant leaves, salt-resilient stems and/or a robust root system to sense, adapt to or even escape salinity, with some of these traits having a close resemblance to mechanisms observed in halophytes. A further recourse is the diverse range of protective metabolites, a strong osmoregulation and a multifaceted antioxidative system that alfalfa can employ under saline conditions. Especially valuable in this context seems to be alfalfa’s ability to flexibly switch defence strategies when facing fluctuating salinity levels. In light of increasing salinity levels and the considerable genetic diversity within and between alfalfa populations, it is imperative to develop alfalfa varieties that have more robust salt-resistance traits and stable breeding lines.

However, the undefined RSA ideotype, the complex genetic architecture of alfalfa’s adaptability to salt stress, the low heritability of salt-tolerant traits in alfalfa along with other open aspects still remain bottlenecks to advance this research field. Identifying stable, key ecophysiological traits underlying the salt-tolerant alfalfa varieties would be a first important step in building the foundation for selective breeding. Moreover, an integrated understanding of the regulation of ecophysiological traits, in conjunction with elucidating the underlying genetic variation and molecular pathways, would be beneficial for advancing alfalfa research. With a more profound understanding of these less well-explored avenues and more and more alfalfa breeding programmes launching, alfalfa is poised to assume a prominent role in combating salinity-related crop losses.
